# The Nuclear Transcription Factor *SlNF-YC9* Regulates the Protrusion of Tomato Fruit Tip

**DOI:** 10.3390/ijms26136511

**Published:** 2025-07-06

**Authors:** Zihan Gao, Ting Long, Pengyu Guo, Junjie Luo, Xiaoqian Nie, Qiaoli Xie, Guoping Chen, Zongli Hu

**Affiliations:** Laboratory of Molecular of Tomato, Bioengineering College, Chongqing University, No. 174 Shazheng Street, Shapingba District, Chongqing 400044, China; gzhfilm@163.com (Z.G.); 202319021067T@stu.cqu.edu.cn (T.L.); guopengyucqu@163.com (P.G.); 20175598@cqu.edu.cn (J.L.); 202319131134@stu.cqu.edu.cn (X.N.); qiaolixie@cqu.edu.cn (Q.X.); chenguoping@cqu.edu.cn (G.C.)

**Keywords:** *SlNF-YC9*, transcription factor, NF-Y complex, fruit protrusion, tomato, plant molecular biology

## Abstract

NF-Y transcriptional regulators play crucial roles in diverse biological processes in plants, primarily through the formation of NF-Y complexes that bind to specific DNA motifs. These complexes modulate the expression of downstream genes, which influence plant development and growth. In our research, the function of the NF-Y family C subunit member *SlNF-YC9* gene in tomato was investigated with the CRISPR/Cas9 method. In contrast to the WT (wild type), the mutant *CR-SlNF-YC9* exhibited a prominent protrusion at the fruit tip. The quantitative PCR analysis displayed that the transcription levels of genes associated with auxin transport (*PIN4*, *PIN5*, and *PIN9*) as well as auxin response genes (*ARF7* and *LAX3*) were enhanced in the *CR-SlNF-YC9* fruits than in the WT. Analysis of dual-luciferase reporter and EMSA assays showed that the SlNF-YC9-YB13b-YA7a trimer specifically binds the *FUL2* promoter and represses its expression. In conclusion, our results suggest that *SlNF-YC9* is crucial in influencing tomato fruit shape by the formation of NF-Y heterotrimeric complexes.

## 1. Introduction

The tomato (*Solanum lycopersicum*) is a vital global crop and serves as a model plant for studying fleshy fruits due to its relatively small genome and established genetic transformation system [1,2]. The shape of the tomato fruit is a key factor influencing fruit quality. Tomatoes have developed diverse fruit sizes and shapes through the process of domestication and improvement, including round, heart, long, flat, rectangular, ellipsoid, and obovoid [3]. The *locule number* (*lc*) and the *fasciated* (*fas*) locus regulate the number of locules in tomato and can directly affect fruit shape and size [4,5]. *SUN* is a key gene controlling the elongated morphology of tomato fruits, and the enhanced transcript level of the *SUN* delivered an effect on the length of tomato fruits [6]. A single mutation in a premature termination codon in the *OVATE* gene results in a transition from round- to pear-shaped fruit [7]. Notably, *SUN* caused an increase by lengthening the number of cells along the entire proximal-distal axis, whereas *OVATE* caused fruit elongation in the proximal region [8]. In heart-shaped fruits, the proximal end is longer than the distal end, resulting in a distinct tip protrusion at the distal end [9].

Previously, there have been relatively few studies on the protuberance of pointed tips in tomato fruits. Overexpression of the *SlFUL2* (*FRUITFULL2*), a MADS-box family transcription factor, induces the formation of tomato fruit pointed tips that may be related to the abnormal style abscission [10]. The loss of function of *PT* (*POINTED TIP*), encoding a C2H2-type zinc finger protein transcription factor, exhibited the promoted formation of pointed-tip fruits by influencing the mRNA abundance of *FUL2* [11]. Recent studies have revealed that an organelle RNA recognition motif (RRM) protein also plays a role in tomato fruit morphology. *CR-slorrm2* exhibits fruit pointed tips at the distal end as well as defective style and ovary development [12]. In addition, the fruit tip protrusion phenotype is observed in both tomato silencing mutants of the gene encoding type B heterotrimeric G protein γ-subunit (*SlGGB1*) [13] and in pyrobactin resistance-like 9 (*PYL9*) silencing mutants [14]. However, the deeper mechanisms underlying the production of fruit tip protrusion are still unclear and need to be explored in further research.

NF-Y (Nuclear transcription factor Y), also known as HAP (Heme-activating protein) or CBFD (CCAAT binding factor domain), is a class of transcription factors widely distributed in both animals and plants [15,16]. NF-Y specifically recognizes and binds to the CCAAT-box within the promoter region of target genes, thereby regulating their transcription [17,18]. NF-Y consists of three distinct subunits: NF-YA (also known as CBF-B or HAP2), NF-YB (CBF-A or HAP3), and NF-YC (CBF-C or HAP5) [19]. NF-YA, NF-YB, and NF-YC subunits typically form large heterotrimeric complexes that function to bind DNA and regulate gene expression [20,21]. During the formation of the NF-Y complex in yeast and animals, NF-YB and NF-YC first form a heterodimer in the cytoplasm and then translocate to the nucleus, where the NF-YA subunits are subsequently recruited to generate the complete complex [22,23]. In yeast and mammals, each subunit of NF-Y is encoded by one (at most two) gene [18]. In contrast, the NF-Y subunits in plants show a familial expansion and are generally encoded by multiple genes encoding the same class of subunits, which creates additional challenges for the study of NF-Y family genes in plants [17,24,25,26,27,28].

Previous studies have shown that NF-Y transcription factors can be extensively involved in plant growth and development [29,30,31,32,33,34,35,36,37]. NF-Y trimeric complexes play crucial regulatory roles across plant species. In *Arabidopsis*, NF-YA/B/C subunits can form various nuclear complexes [25], while the legume MtNF-A1/2-B16-C1/2 trimer controls nodule formation [38]. Similarly, cassava possesses the MeNF-YA1/3-B11/16-C11/12 trimeric complex [39]. Moreover, studies have revealed that NF-Y family genes also play essential roles in regulating plant responses to stress [40,41,42] and photomorphogenesis [43,44]. In tomato, the NF-Y complex consisting of the NF-YB8c-YC1e dimer and NF-YA9, positively regulates the expression of the *SlCHS1* gene, thereby modulating fruit development [45].

So far, in plants, many studies about *NF-Y* genes have been carried out by using *Arabidopsis thaliana* as a model plant, and most research has focused on *NF-YA* and *NF-YB* genes; however, the function of *NF-YC* is not well understood. Hence, it is crucial to investigate the functions of the *NF-YC* genes of tomatoes. In this study, a *SlNF-YC9* knockout vector was constructed and introduced into wild-type tomato using CRISPR/Cas9 technology to produce homozygous *SlNF-YC9* knockout plants. Our data indicated that *CR-nf-yc9* fruits exhibit the tip protrusion phenotype. In addition, this study revealed that *SlNF-YC9* can participate in forming 10 distinct NF-YA-YB-YC9 trimeric complexes. Among them, the SlNF-YC9-YB13b-YA7a trimeric complex was found to suppress the expression of the *SlFUL2* gene. The research elucidates the molecular regulatory mechanisms of *SlNF-YC9* in tomato and fruit apex development, enriches the understanding of the biological functions and pathways of the tomato NF-Y gene family, and provides a theoretical foundation for tomato variety improvement.

## 2. Results

### 2.1. Bioinformatics Analysis, Subcellular Localization, and Expression Pattern of *SlNF-YC9*

**Figure 1:**
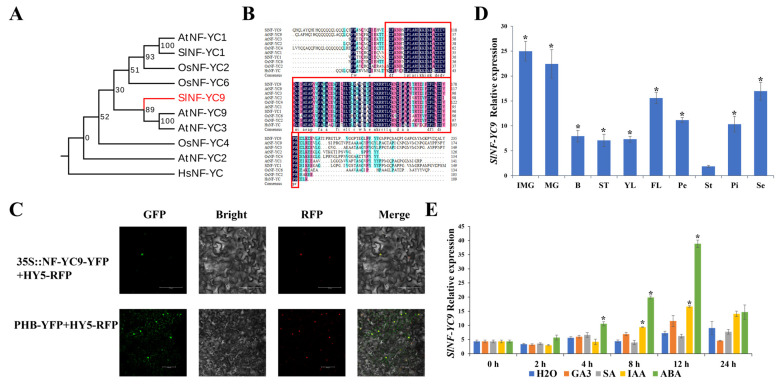
Sequence analysis, subcellular localization, and expression patterns of *SlNF-YC9*. (**A**) *SlNF-YC9* phylogenetic tree. The protein annotation ID are AtNF-YC1 (NP_190428), SlNF-YC1 (XP_004236362), OsNF-YC2 (NC_089037), OsNF-YC6 (NC_089042), *SlNF-YC9* (NP_001233820), AtNF-YC9 (NP_172371), AtNF-YC3 (NP_175880), OsNF-YC4 (NC_089040), AtNF-YC2 (NP_001077726), and HsNF-YC (KAI2516483). (**B**) Homology analysis of *SlNF-YC9*; the red box indicates the CBF domain. The darker the color, the more conserved the amino acid sequence at that position. (**C**) Subcellular localization assay of *SlNF-YC9* protein. YFP: green fluorescent protein; RFP: red fluorescent protein. Red fluorescent protein is used to locate the nucleus. Scale bar = 100 µm. (**D**) Quantitative RT–PCR analysis of the expression of the *SlNF-YC9* gene in immature green (IMG), mature green (MG), breaker (B), stems (ST), young leaves (YL), flowers (FL), petal (Pe), stamen (St), pistil (Pi), and sepals (SE). (**E**) Expression profiles of *SlNF-YC9* induced by GA, SA, IAA, and ABA. Data are means ± SD of three biological replicates. Statistically significant differences were determined using Student’s *t*-test (* *p* < 0.05).

Previous studies have led to the identification of numerous genes in tomato, which have been classified and named based on their amino acid sequence homology [24,45]. We performed a phylogenetic analysis of NF-Y genes in tomato based on available genomic data and the exclusion of several NF-Y genes with highly repetitive sequences. The evolutionary tree results indicated 9 NF-YA genes, 25 NF-YB genes, and 15 NF-YC genes in tomato (Figure S1). Based on the tomato transcript expression data (http://ted.bti.cornell.edu/, accessed on 14 August 2022), NF-YC9, a gene exhibiting relatively high expression levels across various tissues, was selected from the NF-YC gene family as the focus of the study (Figure S2). Phylogenetic tree analysis of *SlNF-YC9* homologous proteins using MEGAX showed that *SlNF-YC9* has the highest homology with *Arabidopsis* NF-YC9 and NF-YC3 (Figure 1A). Multiple protein sequence alignments showed that NF-YC9 proteins are highly conserved across species, all having CBF structural domains (Figure 1B).

To analyze the subcellular localization of the *SlNF-YC9* protein, 35S::SlNF-YC9-YFP fusion vectors were constructed and co-injected with a nuclear localization marker for transient expression in tobacco leaves. The results showed that the green fluorescence emitted by SlNF-YC9-YFP precisely colocalized with the red fluorescence of the nuclear localization marker, suggesting that *SlNF-YC9* proteins are predominantly localized in the nucleus (Figure 1C). To further investigate the function of *SlNF-YC9* in tomato growth and development, we used quantitative real-time PCR (qRT-PCR) to detect the expression levels of *SlNF-YC9* in different tissues and organs of tomato. The results revealed that the transcript levels of *SlNF-YC9* were highest in IMG and MG fruits, followed by flowers, while the lowest expression was found in the stamens of the four floral organs (Figure 1D). Additionally, we analyzed the transcript accumulation of *SlNF-YC9* in seedlings in response to different hormone treatments. The results demonstrated that the expression of *SlNF-YC9* was significantly induced within 8–12 h after IAA and ABA treatments, followed by a rapid decrease (Figure 1E).

### 2.2. The Knockout of *SlNF-YC9* Alters Fruit Morphology in Tomato

To assess the biological function of *SlNF-YC9* in tomato, the CRISPR/Cas9 system was used to generate transgenic tomato plants with *SlNF-YC9* knockout mutants. Two sgRNAs were employed to target the first exon of the *SlNF-YC9* gene for knockout, thereby increasing the knockout success rate. Subsequently, we successfully generated three knockout transgenic lines (*CR-SlNF-YC9*), designated CR-4, CR-8, and CR-9. At the first site of CR-4, there is a 7-bp deletion, and at the second site, a 1-bp deletion; at the first site of CR-9, there is a 5-bp deletion, and at the second site, a 1-bp insertion (Figure 3A). Both of these mutations cause a frameshift, which disrupts the protein function of *SlNF-YC9*. However, at the first site of CR-8, there is a 3-bp deletion, which does not lead to a frameshift mutation. Therefore, we chose CR-4 and CR-9 for subsequent experiments.

**Figure 2:**
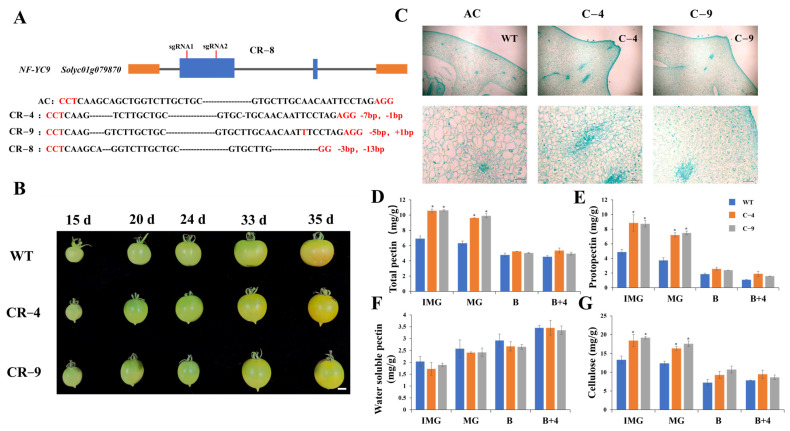
Knockout of *SlNF-YC9* causes tomato fruits with a pointed-tip phenotype. (**A**) The knockout types of *CR-SlNF-YC9*. (**B**) Fruit phenotype of WT and *CR-SlNF-YC9* at 15, 20, 24, 33, and 35 days (post-anthesis). Scale bar: 1 cm. (**C**) Distal longitudinal section of *CR-SlNF-YC9* transgenic lines and wild-type fruit at 15 days. Scale bar: 1 mm. (**D**–**G**) Physiological parameter measurements of WT and *CR-SlNF-YC9* at four different stages. (**D**) Total pecStin content, (**E**) protopectin content, (**F**) water soluble pectin content, and (**G**) cellulose content. Data are means ± SD of three biological replicates. Statistically significant differences were determined using Student’s *t*-test (* *p* < 0.05).

Subsequently, we observed that *CR-SlNF-YC9* exhibited a noticeable protrusion at the fruit distal end compared to the wild type (WT) (Figure 2B). Moreover, anatomical analysis of the *CR-SlNF-YC9* fruits was performed using paraffin-embedded sections. The results revealed that there was no obvious shedding zone in the pointed tips of the *CR-SlNF-YC9* fruits, and notably denser cell arrangement and smaller cell size were observed in *CR-SlNF-YC9* compared to the WT (Figure 2C). Furthermore, the pectin and cellulose content in fruits at different developmental stages of *CR-SlNF-YC9* were measured. (Figure 2D–G). The results showed that the protopectin, total pectin, and cellulose contents in the knockout lines were significantly higher than those in the WT during the IMG and MG stages (Figure 2D,E,G), while the water-soluble pectin content showed no significant changes (Figure 2F). Furthermore, the qRT-PCR results showed that there were corresponding changes in the *CR-SlNF-YC9* mutants in genes involved in cell expansion (*EXP1*), cell wall structure modification (*XTH2* and *XTH3*), and cell cycle regulatory factors (*Cyc B1;1* and *CycD3;1*) (Figure S3). Taken together, these results provide evidence that knockout of *SlNF-YC9* can affect the protrusion formation of tomato fruit tips.

### 2.3. The Knockout of *SlNF-YC9* Affects the Expression Levels of the FUL2 Gene in Tomato Fruits

**Figure 3:**
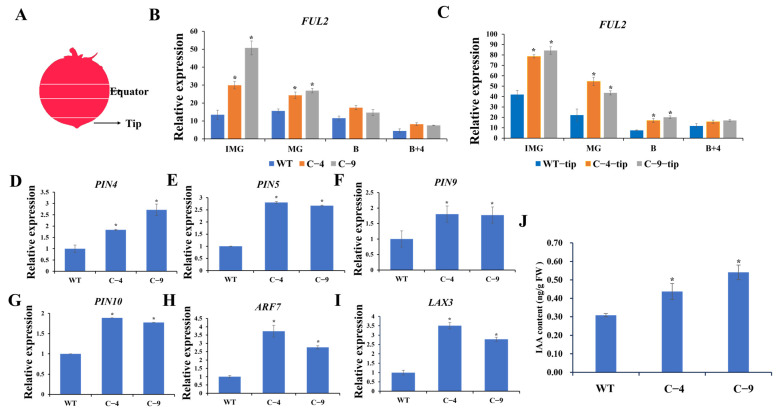
Expression levels of *FUL2*, auxin-related genes, and IAA content measurement (**A**) Schematic representation of the apical and equatorial parts of the tomato fruit. (**B**,**C**) Expression levels of *FUL2* in *CR-SlNF-YC9* and WT at four different times were determined using qRT-PCR. (B) Expression of *FUL2* in the fruit equators, (**C**) Expression of *FUL2* in fruit tips. (**D**–**I**) Expression levels of auxin-related genes in *CR-SlNF-YC9* and WT, including (**D**) *PIN4*, (**E**) *PIN5*, (**F**) *PIN9*, (**G**) *PIN10*, (**H**) *ARF7,* and (**I**) *LAX3*. (**J**) Endogenous IAA content in WT and *CR-SlNF-YC9* fruits (IMG period fruits). Data are means ± SD of three biological replicates. Statistically significant differences were determined using Student’s *t*-test (* *p* < 0.05).

To investigate the regulatory mechanism of the *SlNF-YC9* gene in fruit tip projection, the expression of several fruit morphogenesis genes in WT and *CR-SlNF-YC9* fruits was analyzed. It was found that among these genes, only the expression levels of *FUL2* were significantly changed (Figure 3B), while the transcript accumulation of the other genes (*SUN*, *OVATE*, *PT,* and *SlORRM2*) involved in the development of fruit shape had no significant changes compared to the WT (Figure S4). Moreover, the mRNA abundance of *FUL2* at different developmental stages and in different fruit parts was examined. According to our results, the transcript levels of *FUL2* gradually decreased along with fruit ripening, and the expression levels of *FUL2* were significantly up-regulated in *CR-SlNF-YC9* at the IMG period compared with the WT (Figure 3B,C). In addition, the mRNA levels of *FUL2* were higher in the fruit tip than in the fruit equator at all four fruit periods, suggesting that *SlNF-YC9* primarily affects the protrusion of the fruit tip at the distal end of the fruit, rather than the entire fruit.

### 2.4. The Knockout of *SlNF-YC9* Affects the IAA Content and Expression of Auxin-Related Genes in Tomato Fruits

Previous studies have demonstrated that the development of the fruit tips in *FUL2*-OE was regulated through the auxin pathway [45]. Therefore, the expression levels of auxin-related genes in *CR-SlNF-YC9* and WT fruits were examined. The qRT-PCR results showed that the transcript levels of *PIN4, PIN5, PIN9*, and *PIN10* genes, which are crucial for auxin transport, in the fruit tips of *CR-SlNF-YC9* were significantly higher than those in the WT (Figure 3D–G), whereas *FZY1*, *FZY2*, *FZY5,* and *FZY6* genes associated with IAA synthesis pathways showed no significant differences when compared to the WT (Figure S5). To validate these results, we quantified endogenous IAA levels and observed that the IAA content in fruit tips of *CR-SlNF-YC9* was higher than the WT (Figure 3J). Based on the results of the IAA-induced expression profile (Figure 1E), the IAA-responsive genes were also analyzed. The qRT-PCR results showed that *ARF7* and *LAX3* were significantly upregulated in *CR-SlNF-YC9* compared to the WT (Figure 3H,I).

### 2.5. RNA Sequencing Analysis of CR-*SlNF-YC9* Fruit Tips

**Figure 4:**
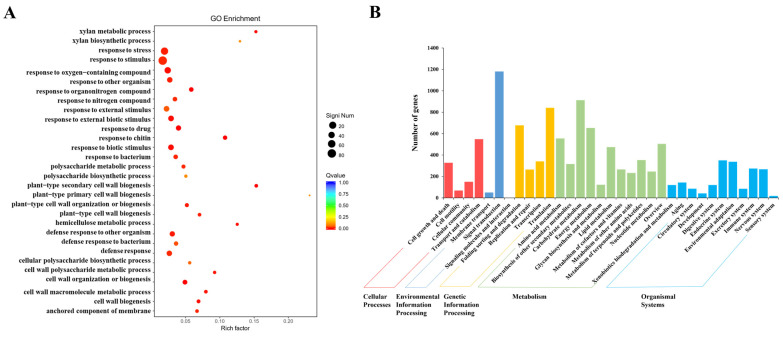
RNA-seq analysis of *CR-nf-yc9* fruits (**A**) GO classification. (**B**) KEGG pathway enrichment analysis. Genes differentially expressed in *CR-nf-yc9* and WT fruit. With such a small sample size, the analysis results would likely exhibit high randomness and lack practical significance.

**Figure 5:**
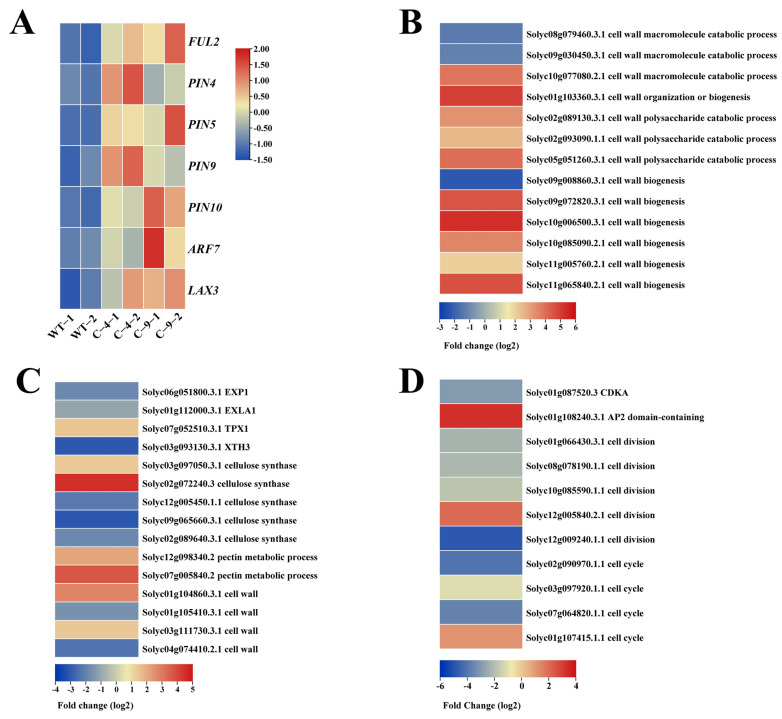
Differentially expressed gene analysis in transcriptome sequencing of *CR-SlNF-YC9* fruits. (**A**) Heatmap showing changes in growth hormone-related gene expression through transcriptome sequencing. Heatmaps show changes in the expression of genes associated with cell wall synthesis, cellulase (**B**), pectin cell expansion, cell wall (**C**), cell division, and cell cycle signaling (**D**). Heatmap based on differential gene log_2_ values, scale bar represents fold change in differential gene log_2_.

To enhance the investigation of the molecular mechanism of tomato fruit tips development, a transcriptome analysis of the WT and *CR-SlNF-YC9* fruit tips was performed. A total of 959 differentially expressed genes were identified in the knockout mutant compared to the WT, of which 406 were upregulated and 553 were downregulated (Figure S6). To further categorize the differentially expressed genes, we performed Gene Ontology (GO) enrichment analysis and Kyoto Encyclopedia of Genes and Genomes (KEGG) Pathway classification on all differentially expressed genes. Functional annotation analysis showed that knockout of *SlNF-YC9* was involved in cell wall biosynthesis, metabolism, auxin polar transport, and regulation of stimulus responses (Figure 4A). In terms of biological processes, the differentially expressed genes were mainly distributed in metabolic and cellular processes, and in terms of molecular functions, the differentially expressed genes were mainly distributed in the areas of catalytic activity and binding activity (Figure 4B). To further validate our results, we focused on growth hormone pathway-related genes and found that the RNA-seq data were consistent with our quantitative results (Figure 5A). Given the more densely arranged and smaller cell morphology of *CR-SlNF-YC9* fruit tip cells (Figure 2C), relevant genes involved in cell division, cell cycle, and cell wall organization processes were also focused on. Transcript levels of genes related to cell wall catabolism, such as expansin (EXP), xyloglucan endotransglucosylase hydrolase (XTH), cellulase synthetase, and pectin methyl esterase (PME), were relatively significantly altered in the mutants (Figure 5B,C). Furthermore, the expression of genes related to cell wall biosynthesis, cell division, and cell cycle also changed significantly in the mutants (Figure 5D). These results suggest that *SlNF-YC9* may further influence the protrusion of fruit tip formation through the auxin and cell wall pathway.

### 2.6. *SlNF-YC9* Can Form Multiple Trimeric Complexes with SlNF-YBs and SlNF-YAs

**Figure 6:**
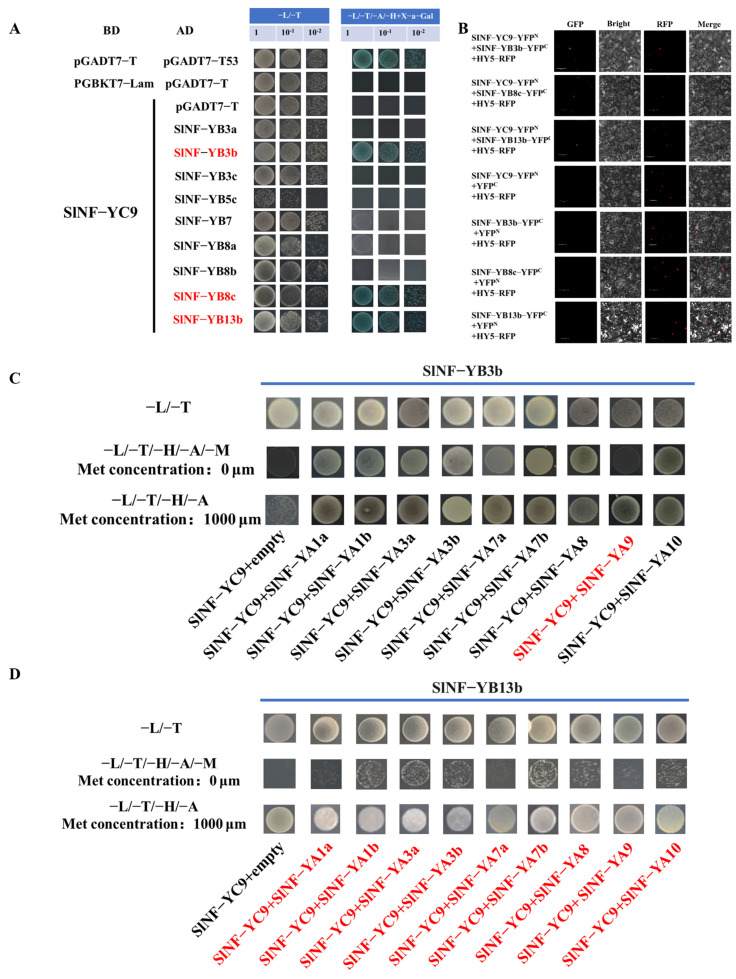
*SlNF-YC9* protein forms dimeric complexes with SlNF-YB3b, SlNF-YB8c, and SlNF-YB13b, and Yeast three-hybrid assays. (**A**) The protein-protein interactions between *SlNF-YC9* and SlNF-YBs, respectively, were examined using a yeast two-hybrid study. Positive control: pGBKT7-53 + pGADT7-T; negative control: pGBKT7-Lam + pGADT7-T. Red markers indicate that *SlNF-YC9* can interact with these SlNF-YBs. (**B**) Interaction of *SlNF-YC9* with SlNF-YB3b, SlNF-YB8c, and SlNF-YB13b by bimolecular fluorescence complementation assays. HY5-RFP, a nuclear localization control. RFP, red fluorescent protein; YFP, yellow fluorescent protein. Scale bars, 50 μm. (**C**) Yeast three-hybrid hybridization assays to detect interactions between SlNF-YAs proteins and SlNF-YC9-Yb3b heterodimers. (**D**) Yeast three-hybrid hybridization assays to detect interactions between SlNF-YAs proteins and SlNF-YC9-Yb13b heterodimers. The double dropout medium (-L-T) were used as a control; The main difference between the quadruple (-L/-T/-H/-A/-M) and quintuple (-L/-T/-H/-A) deficient mediums were the presence or absence of methionine, and the presence of differences in the growth of yeast in the quadruple and quintuple deficient mediums represents an interplay between the three proteins. Labeling SlNF-YA, which can participate in the formation of trimeric complexes, in red color.

The NF-YB and NF-YC subunits typically form dimers, which then form heterotrimeric complexes upon recruitment of NF-YA, which then recognize the CCAAT element that binds to the promoter of the target gene, thereby regulating the transcription of the target gene [16,25]. Therefore, we speculate that it is also possible that the formation of the fruit tip projection is further regulated in *CR-SlNF-YC9* by the formation of the NF-Y complex. Nine SlNF-YBs proteins with relatively high expression in tomato fruits were selected by tomato transcript expression data, followed by protein-protein interaction experiments to screen for SlNF-YBs proteins capable of forming SlNF-YB-YC9 complexes. These SlNF-YBs-AD were co-expressed with SlNF-YC9-BD in yeast cells, revealing that only SlNF-YB3b-AD, SlNF-YB8c-AD, and SlNF-YB13b-AD could be grown on a quadruple dropout medium (SD/-Leu-Trp-His-Ade+X-a-gal), similar to the yeast cells harboring pGADT7-T and pGBKT7-53 (positive control) (Figure 6A). The BiFC assay results showed that stronger fluorescent signals were detected in the epidermal cell nucleus of N. benthamiana leaves only when SlNF-YC9-YFPN was co-expressed with SlNF-YB3b-YFPC, SlNF-YB8c-YFPC, and SlNF-YB13b-YFPC (Figure 6B). These results demonstrate that *SlNF-YC9* was able to interact with SlNF-YB3b, SlNF-YB8c, and SlNF-YB13b to form dimers.

After screening for three different SlNF-YB proteins that form dimers with *SlNF-YC9*, further screening was performed to identify SlNF-YA subunits recruited by the NF-YB-YC dimer. These SlNF-YA proteins, obtained from our evolutionary tree analysis of NF-YA (Figure S1) in tomato, were subjected to yeast three-hybrid experiments with SlNF-YB3b, SlNF-YB8c, SlNF-YB13b, and *SlNF-YC9*, respectively (a total of 27 different combinations, Table S1). SlNF-YAs and *SlNF-YC9* were inserted into the pBridge vector to construct the SlNF-YC9-YA expression vector, which was co-transformed with the SlNF-YBs-pGADT7 expression vector into yeast for Y2H progression. The yeast was then grown on double (SD/-Leu-Trp), quadruple (SD/-Leu-Trp-His-Ade), and quintuple (SD/-Leu-Trp-His-Ade-Met) dropout media. The yeast co-transformed with the SlNF-YC9-YB13b dimer and all SlNF-YAs grew normally on media with high concentrations of methionine but failed to grow in methionine-free media. However, the yeast co-transformed with SlNF-YC9-YB3b and SlNF-YA9 failed to grow in methionine-free media (Figure 6C,D). In addition, the yeast co-expressing the SlNF-YC9-YB8c dimer with all SlNF-YAs grew normally at different concentrations of methionine (Figure S7A, B). The results indicated that SlNF-YC9-YB3b forms a heterotrimer with SlNF-YA9, and NF-YC9-YB13b forms heterotrimers with SlNF-YA1a, SlNF-YA1b, SlNF-YA3a, SlNF-YA3b, SlNF-YA7a, SlNF-YA7b, SlNF-YA8, SlNF-YA9, and SlNF-YA10, respectively. While the SlNF-YC9-YB8c dimer cannot form any intact trimeric complexes with SlNF-YAs.

### 2.7. SlNF-YC9-YB13b-YA7a Trimeric Complex Represses the Promoter Activity of FUL2

**Figure 7:**
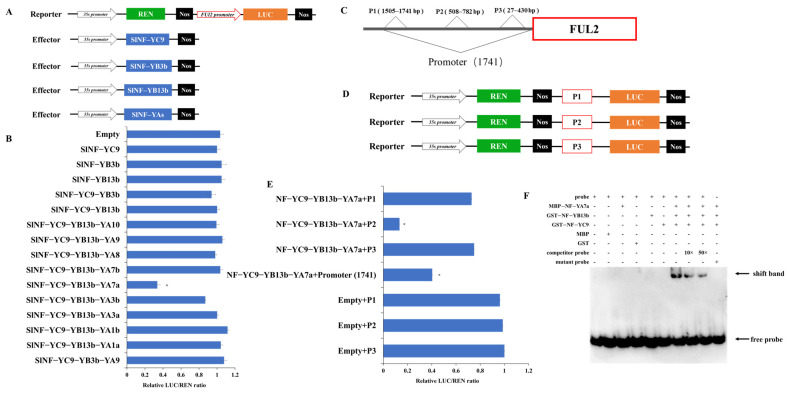
SlNF-YC9-YB13b-YA7a directly regulates the transcriptional activity of the *FUL2* gene. (**A**) Schematic representation of the effector and reporter structures used in transient expression in the dual-luciferase assays. (**B**) Dual-luciferase assays to analyze the ability of individual NF-Y subunits or NF-Y complexes to affect the *FUL2* promoter activity. Empty indicates pGreenII 62-SK empty vector. (**C**) Schematic representation of different regions in the *FUL2* promoter. (**D**) Schematic representation of reporter structures used in transient expression in the dual-luciferase assays. (**E**) The influence of SlNF-YC9-YB13b-YA7 complex on the transcription activity of different regions of the *FUL2* promoter. Empty indicates pGreenII 62-SK empty vector. The LUC/REN ratio at no load is set to 1 as a standard value. (**F**) EMSA shows that the SlNF-YC9-YB13b-YA7 trimer specifically binds the CCAAT-box motif. Data are means ± SD of three biological replicates. Statistically significant differences were determined using Student’s *t*-test (* *p* < 0.05).

The *CR-SlNF-YC9* fruits exhibited similar phenotypes of fruit tip protrusion as the *FUL2*-OE fruits [10], and *FUL2* was significantly upregulated in the *CR-SlNF-YC9* fruits (Figure 2B). Previous studies have shown that the NF-Y complex can regulate the transcriptional activity of downstream target genes by binding to the CCAAT element in the promoter region [45]. Moreover, five CCAAT elements were found in the *FUL2* promoter sequence (Table S2). Therefore, we hypothesized that the *SlNF-YC9* alone, or SlNF-YC9-YB dimers, or SlNF-YC9-YB-YA heterotrimers may directly regulate the expression of the *FUL2* gene. Dual-luciferase reporter assays were performed. The effector vectors containing the coding sequences of *SlNF-YC9*, SlNF-YB3b, SlNF-YB13b, and SlNF-Yas, respectively, and the reporter vector containing *FUL2* promoter sequences (1741 bp upstream of the transcriptional start site) (Figure 7A), were transiently expressed in tobacco leaves via *Agrobacterium*-mediated infiltration, according to the different combinations shown in Figure 7B. Dual-luciferase assays revealed that only the complex formed by SlNF-YC9-YB13b-YA7a significantly inhibited *FUL2* promoter activity in vivo (Figure 7A,B), whereas the other eight SlNF-YC9-YB13b-YAs trimeric complexes, the individual NF-Y proteins (*SlNF-YC9*, SlNF-YB3b, SlNF-YB13b), the SlNF-YC9-YB3b and SlNF-YC9-Y13b dimers did not affect the promoter activity of *FUL2*.

To verify the specific binding site of the SlNF-YC9-YB13b-YA7a complex to the *FUL2* promoter, the reporter vectors were categorized into three types (Figure 7C,D): P1 includes sequences from 1505–1741 bp upstream of the transcription start site (containing two CCAAT binding sites); P2 includes sequences from 508–782 bp upstream of the transcription start site (containing one CCAAT binding site); P3 includes sequences from 27–430 bp upstream of the transcription start site (containing two CCAAT binding sites). The relative LUC/REN ratios in co-transformed tobacco leaves carrying the effectors *SlNF-YC9*, YB13b, YA7a, and the P2 reporter plasmid were significantly downregulated compared to the control (Figure 7E). To further confirm the direct binding of the SlNF-YC9-YB13b-YA7 trimer to the *SlFUL2* promoter, this study performed EMSAs using biotin-labeled oligonucleotides containing the CCAAT motif. The results demonstrate that when a mixture of the three tomato transcription factor proteins (*SlNF-YC9*, SlNF-YB13b, and SlNF-YA7) was incubated with the biotin-labeled double-stranded DNA probe harboring the CCAAT cis-element, a clear mobility shift was observed (Figure 7F). The results suggest that the SlNF-YC9-YB13b-YA7a complex may inhibit the expression of the *FUL2* gene by binding to the P2 region of the *FUL2* promoter.

## 3. Discussion

The NF-Y family is an important class of multi-subunit nuclear transcription factors that are widely involved in the regulation of gene expression in plants. Members of the NF-Y family play a crucial role in various physiological processes during plant growth and development, including seed germination, root development, and flower formation [46,47]. In tomato, virus-induced gene silencing (VIGS) is used to suppress the NF-Y genes, and five NF-Y genes were found to affect fruit ripening [24]. In our study, the *CR-nf-yc9* mutant showed no changes in fruit ripening but did show a phenotype in fruit tip development (Figure 2B). Some genes affecting fruit shape development, such as *SUN*, *OVATE*, and *PT*, showed no significant differences in expression levels in *CR-SlNF-YC9* fruit tips compared to WT. (Figure S4). Notably, we found that the mRNA abundance of *FUL2* in *CR-nf-yc9* was significantly upregulated (Figure 3B,C), which corresponds to the past studies that overexpression of *FUL2* can cause protrusion of tomato fruit tips [10,11]. Based on these results, we hypothesized that the regulatory mechanism of *SlNF-YC9* in tomato fruit tip protrusion is likely to be connected to the *FUL2* gene.

NF-Y family genes are widely found in plants and animals and play important roles as transcription factors in the regulation of gene expression. They usually consist of three subunits: NF-YA, NF-YB, and NF-YC [48]. The three subunits interact with each other, collectively recognizing and binding to specific DNA sequences, thereby regulating the transcriptional activity of target genes [18,49]. Previous studies have shown that the assembly process of NF-Y heterotrimer is strictly ordered. NF-YB and NF-YC assemble into dimers outside the nucleus via histone folding structural domains. Subsequently, inside the nucleus, the A1 (α-helix) of the NF-YA subunit tightly binds to the surface grooves to form the NF-Y heterotrimer [49]. The assembly of the NF-Y complex in plants follows the same mechanism. In tomato, the complete NF-Y complex consisting of NF-YB8c-YC1e dimer and NF-YA9 positively regulates the expression of the *SlCHS1* gene and specifically binds to the CCAAT element upstream of the *SlCHS1* gene promoter [45]. Moreover, NF-YA7 can interact with NF-YB2-YC4 dimer to form a heterotrimer, which in turn is involved in regulating the response of Brassica napus under drought stress [50]. In our study, we screened SlNF-YC9-YB3b-YA9, SlNF-YC9-YB13b-YA1a/1b/3a/3b/7a/7b/8/9/10, a total of 10 different trimeric complexes, by yeast two-hybrid, BiFC (Figure 6A,B), and three-hybrid experiments (Figure 6C,D). The CCAAT motif is a typical promoter element widely found in the promoter regions of many genes in eukaryotes [51,52,53]. The NF-Y complex recognizes and specifically binds to the sequence through its unique DNA-binding ability, thereby regulating the transcriptional expression of downstream genes [21,54,55]. In this research, dual luciferase experiments showed that neither *SlNF-YC9* alone nor the complexes formed by *SlNF-YC9* and SlNF-YBs could affect *FUL2* transcription (Figure 7B). However, only the SlNF-YC9-YB13b-YA7a complex was able to inhibit *FUL2* expression (Figure 7B,E). Our study provided insights into the many different forms of NF-Y family complexes in tomato, revealing the diversity and specificity of these complexes in the regulation of gene expression.

Plant hormones are pivotal in plant growth and development, and the NF-Y family also plays a critical role in the regulation of plant hormones. In *Arabidopsis*, the *NF-YC3/4/9* genes can inhibit GA-regulated seed germination; at the protein level, they were able to interact with the DELLA protein RGL2 to inhibit *ABI5* expression [56]. Overexpression of the *CdtNF-YC1* gene in rice significantly enhances the expression of ABA response, synthesis, and signal genes and improves plant drought tolerance [41]. In our study, *SlNF-YC9* affected the expression levels of several IAA transporter genes in fruit tips (Figure 3E–H). Additionally, the hormone expression pattern of *SlNF-YC9* indicated its responsiveness to IAA (Figure 1E), and IAA-responsive genes were also significantly upregulated in *CR-SlNF-YC9* (Figure 3I,J). Subsequently, significantly higher IAA content was detected in fruits of *SlNF-YC9* knocked out compared to the WT (Figure 3D). The above results suggest that *SlNF-YC9* plays an important function in auxin transport and response.

Cell wall metabolism is crucial in plant development, involving the synthesis, degradation, and remodeling of cell wall components, which directly affect plant structure and morphology. During tomato fruit development, pectin and cellulose play key roles. Their interaction regulates the mechanical properties of the cell wall, thereby influencing the fruit’s size, shape, firmness, and market quality [57,58,59,60,61,62]. In our analysis, *CR-SlNF-YC9* fruits contained significantly higher levels of protopectin, total pectin, and cellulose than WT (Figure 2D,E,G). Additionally, a large number of changes in cell wall metabolism, pectin, and cellulase-related gene expression were detected in *CR-nf-yc9* by RNA-seq (Figure 5B–D). Notably, the key gene *EXP1* involved in cell expansion showed a significant decrease in *CR-SlNF-YC9*, corresponding to which paraffin-embedded experiments revealed that knockout of *SlNF-YC9* reduced cell size in tomato fruit tips. (Figure 2C). The above results suggest that *SlNF-YC9* may affect the shape of tomato fruits by influencing cell expansion and pectin and cellulose metabolism, but further experiments are needed to verify this.

**Figure 8:**
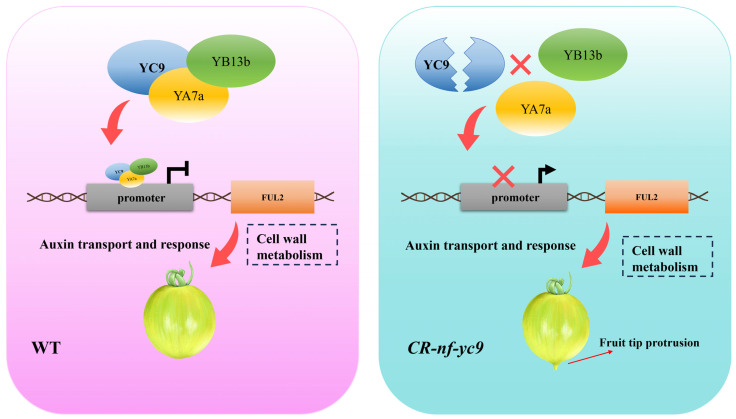
Proposed model depicting the role of *SlNF-YC9* in tomato fruit tip protrusion. Dashed lines indicate possible regulatory mechanisms.

Taken together, our study shows that a rather pronounced bulging of the fruit tip occurs in *CR-nf-yc9*. The mechanistic analysis suggests that the knockout of *SlNF-YC9* prevents the formation of the complete SlNF-YC9-YB13b-YA7 complex in tomato plants, thereby relieving the repressive effect of the SlNF-Y complex on the expression of the *FUL2* gene. This enhances the expression of the *FUL2* gene and subsequently promotes the formation of the fruit tip protrusion by the auxin signaling pathway. In addition, *SlNF-YC9* may also affect fruit development through the cell expansion, cell wall metabolism pathway (Figure 8). In conclusion, our study enriches the regulatory network of tomato fruit shape and also further uncovers the biological functions of *SlNF-Y* transcription factors in plants.

## 4. Materials and Methods

### 4.1. Plant Materials and Growth Conditions

The study involved wild-type tomato (*Solanum lycopersicum Mill cv. Ailsa Craig, AC++*), *CR-SlNF-YC9* transgenic plants, along with *Nicotiana benthamiana*. The plants were grown under standard conditions: daytime temperature (26 °C), nighttime temperature (18 °C), 16 h of light and 8 h of darkness, 70% relative humidity, and a light intensity of 250 μmol m^−2^ s^−1^.

### 4.2. Bioinformatics Analysis

The sequence information of *SlNF-YC9* was acquired from the SGN and NCBI websites, accessed on 2 October 2020. The evolutionary tree depicted in this article was generated using MEGA 6.06 and EVOLVIEW software V2. The DNAMAN 5.2.2 tool was employed to perform the alignment of several sequences between *SlNF-YC9* and its family proteins. We analyzed the *FUL2* promoter using plantCARE (Plant promoter Database, accessed on 2 June 2022).

### 4.3. Subcellular Localization Assay

The entire coding sequence of *SlNF-YC9*, with the termination codon removed, was amplified and fused with GFP to create the PHB-eGFP expression vector. The engineered vector was introduced into *Agrobacterium* GV3101, then transferred to tobacco via *Agrobacterium*-mediated genetic transformation. Following a 2-day period of darkness treatment, the fluorescence signal was detected using a confocal microscope. HY5-RFP was used as a nuclear localization marker [63]. The primers utilized for constructing the vector are displayed in Table S3.

### 4.4. *SlNF-YC9* Knockout Vector Construction and Plant Transformation

The CRRISPR-GE (http://skl.scau.edu.cn/home/, accessed on 1 October 2020) website was used to select a knockout target on the first exon sequence of *SlNF-YC9*, and then construct the CRISPR/Cas9-*SlNF-YC9* vector. The vector was introduced into the Agrobacterium strain LBA4404 and then transferred to the wild-type tomato by the Agrobacterium-mediated genetic transformation method. Transgenic plants were screened on a resistant medium containing kanamycin at a concentration of 50 mg L^−1^. Subsequently, the gene sequence of *SlNF-YC9* was analyzed through sequencing to identify the specific type of knockout. The homozygous knockout transgenic lines were used for further investigation. The primers used for vector construction are shown in Table S3.

### 4.5. Measurement of Pectin and Cellulase Content

Determination of pectin content: The samples (Fruit tips of 20 days post-anthesis) were ground in liquid nitrogen. A 0.2 g portion of material was weighed, mixed with 1 mL of 95% ethanol, and placed in a drying oven at 95 °C for 30 min. After cooling to room temperature, the mixture was centrifuged at 8000 rpm for 5 min, and then the supernatant was discarded. Then, 1 mL of water was added to the precipitate, and the mixture was placed in a 50 °C water bath for 30 min. After cooling, the mixture was centrifuged at 8000 rpm for 5 min, and the supernatant was discarded (the supernatant can be used for the determination of water-soluble pectin). Next, 1 mL of extract was added to the precipitate, mixed well, and placed in a 95 °C water bath for 1 h. After cooling, the mixture was centrifuged at 8000 rpm for 10 min, and the supernatant was collected for the determination of original pectin. The formula is as follows: pectin content (mg/g) = (C × V) × ΔA2_530_/ΔA1_530_/(W × V1/V2). The formula ΔA1_530_ represents the difference between the absorbance values of the standard tube and the blank tube at 530 nm; ΔA2_530_ represents the difference between the absorbance values of the measurement tube and the control tube at 530 nm; C is the standard sample concentration (mg/mL); V is the volume of the standard added to the reaction system (mL); V1 is the volume of the sample added to the reaction system (mL); V2 is the volume of extract (mL); W is the sample’s fresh weight (g).

Determination of cellulase content using the Cellulose Extraction kit (Michy Biology, Suzhou, China). The samples were ground in liquid nitrogen, 0.1 g of material was weighed, mixed with 1 mL of 80% ethanol, placed in an 80 °C water bath for 30 min, cooled down, and then centrifuged at 8000 rpm for 5 min, with the supernatant being discarded. Then 1 mL of acetone was added, vortexed for 2 min, centrifuged at 8000 rpm for 5 min, and the supernatant was discarded. Then 1 mL of reagent I was added, vortexed and shaken for 2 min, soaked for 15 h, and then centrifuged at 8000 rpm for 5 min, and the supernatant was discarded. After washing twice with distilled water, the precipitate was dried. Finally, 1 mL of reagent III was added to the precipitate and mixed well, then centrifuged at 10,000 rpm for 10 min, and the supernatant was taken to measure the absorbance at 620 nm. The free cellulase content was calculated using the following formula: Cellulase content (mg/g) = [(ΔA_620_ − 0.0194)/(2.8737 × V1)]/(W × V1/V2) × Dilution Factor, where ΔA_620_ is the difference between the absorbance values of the measurement tube and the blank tube at 530 nm; V1 is the volume of samples (mL); V2 is the volume of extract (mL); W is sample fresh weight (g).

### 4.6. Fruit Tip Tissue Sections

Fruit tips of WT and *CR-SlNF-YC9* were collected 15 days post-anthesis, treated with 1% hydrochloric acid for 10 min, and then placed in 70% FAA fixative for 24 h and embedded in Paraplast Plus (Sigma, Saint Louis, Missouri, USA). Longitudinal sections of the fruit tips were stained briefly with 0.04% (*w*/*v*) toluidine blue solution. After that, the samples were examined with a dissecting microscope (Nikon E100).

### 4.7. qRT-PCR Analysis and RNA-Sequencing

Total RNA was extracted from samples (Fruit tips of 20 days post-anthesis) using the Takara RNAiso Plus reagent. The M-MLV Reverse Transcriptase kit (Promega, Beijing, China) was used to convert RNA samples into cDNA. According to the manufacturer’s instructions, the transcription levels of specific genes were quantitatively analyzed using the SYBR Premix Ex Taq II kit (Takara, Ōtsu, Shiga, Japan) and gene-specific primers, using *SlCAC* (*Solyc08g006960*) as an internal reference gene. Quantitative reverse-transcription-PCR (qRT-PCR) was carried out using the CFX Connect Real-Time System (Bio-Rad, Hercules, California, USA). The analysis of relative expression levels was conducted using the 2^−ΔΔCT^ method. Primers used in reverse transcription and qPCR are listed in Table S3.

### 4.8. RNA Sequencing

Fruits of the *CR-SlNF-YC9* and WT were harvested 15 days after anthesis. The apices were collected from the distal end of the fruit and immediately frozen in liquid nitrogen. Samples were collected in three biological replicates and sent to Sangon Biotech (Shanghai, China) for RNA-seq analysis. Differentially expressed genes (DEG) with more than twofold change in expression level with *p* value < 0.05 were screened out in Table S4.

### 4.9. Yeast Two-Hybrid and BiFC Assays

Yeast two-hybrid assays: The bait plasmid pGBKT7-*SlNF-YC9* and the prey plasmid pGADT7-SlNF-YC3a, 3b, 3c, 5c, 7, 8a, 8b, 8c, and 13b were co-transformed into yeast strain Y2HGold. The transformed yeast was then plated on the SD medium lacking tryptophan and leucine, followed by incubation for 3 days. Thereafter, a single colony was selected and inoculated on the SD medium devoid of tryptophan, histidine, adenine, and leucine. This plate was incubated upside down for 1–2 days [64].

BiFC assays: The recombinant SlNF-YC9-YFPN, SlNF-YB3b-YFPC, SlNF-YB8c-YFPC, and SlNF-YB13b-YFPC vectors were transformed into the *Agrobacterium tumefaciens* GV3101. Then, SlNF-YC9-YFPN, SlNF-YB3b-YFPC, SlNF-YB8c-YFPC, SlNF-YB13b-YFPC, and control HY5-RFP were infiltrated into 4-week-old tobacco leaves by *Agrobacterium*-mediated genetic transformation. The subsequent experimental steps refer to the methods in the subcellular localization assay [65]. The primers used to construct the vector are shown in Table S3.

### 4.10. Yeast Three-Hybrid

Based on the principles of yeast two-hybrid experiments, *SlNF-YC9* was inserted into the MCS I site (Bait site) of the pBridge vector, and SlNF-YAs were inserted into the MCS II site (Bridge site, Met-sensitive site) of the pBridge vector. SlNF-YBs were inserted into the pGADT7 vector. The constructed pGADT7 and pBridge plasmids were then co-transfected into the Y2HGold yeast. The transformed yeast cells were cultured on double (SD/-Leu-Trp), quadruple (SD/-Leu-Trp-His-Ade), and quintuple (SD/-Leu-Trp-His-Ade-Met) dropout media. The plates were incubated upside down for 1–2 days. The primers used to construct the vectors are shown in Table S3.

### 4.11. Transient Expression Assays

The coding sequences of *SlNF-YC9* and SlNF-YB3b and SlNF-YB13b and SlNF-YA 1a, 1b, 3a, 3b, 7a, 7b, 8, 9, 10, were introduced into pGreenII 62-SK for effector, and promoter fragments of *FUL2* were cloned into the pGreenII 0800-LUC vector as reporters, respectively. After the introduction of the recombinant plasmid into Agrobacterium GV3101, the recombinant plasmid was infiltrated into tobacco leaves. Post-infiltration with Agrobacterium, the leaves were stored in the dark for 1 day, followed by 2 days under light. The activities of LUC and REN were determined by using an enzyme marker according to the instructions of the Promega Dual-luciferase assay kit, and the ratio of LUC/REN was calculated [66].

## Data Availability

Data are contained within the article or Supplementary Materials.

## Supplementary Materials

The following supporting information can be downloaded at: https://www.mdpi.com/article/10.3390/ijms26136511/s1.
