# Supplementary material for: The Nuclear Transcription Factor SlNF-YC9 Regulates the Protrusion of Tomato Fruit Tip
Source: Int J Mol Sci. 2025 Jul 6;26(13):6511. doi: 10.3390/ijms26136511 (PMC12249496; doi:10.3390/ijms26136511)
Supplement: Supplementary file 1 [file ijms-26-06511-s001.zip › Supplement Figures S1-S7.pdf]

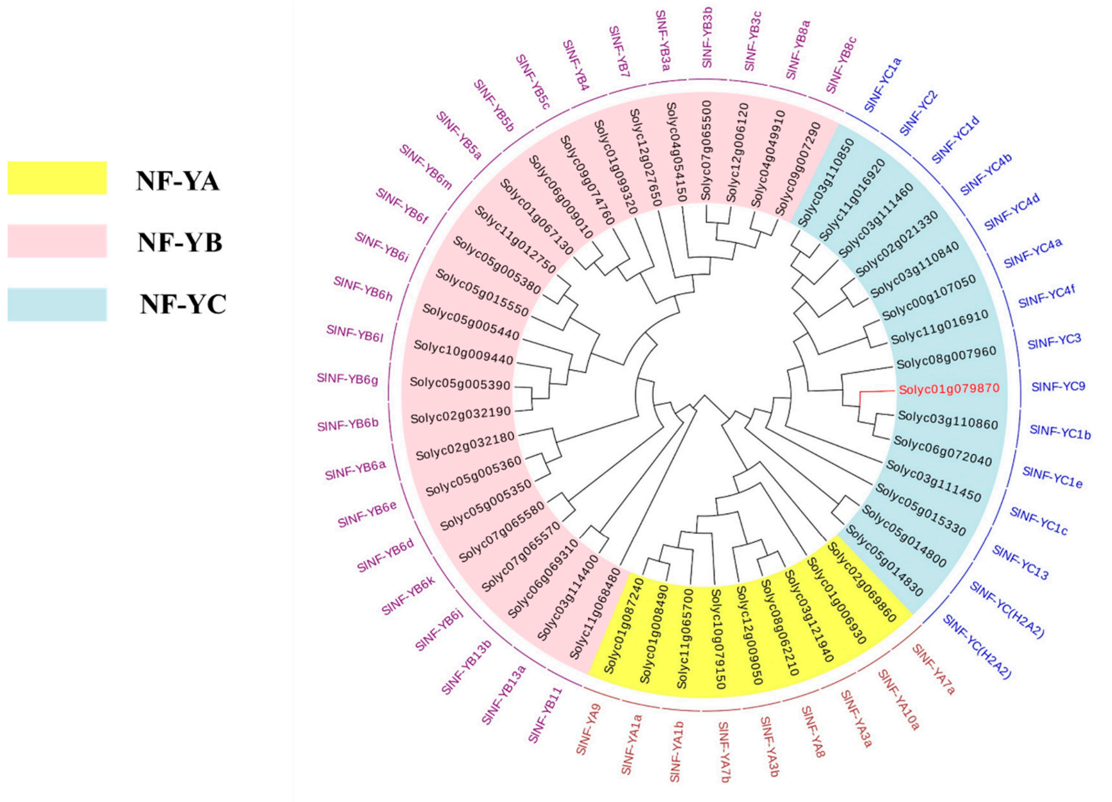

Figure S1. Evolutionary tree analysis of NF-Y family genes in tomato.

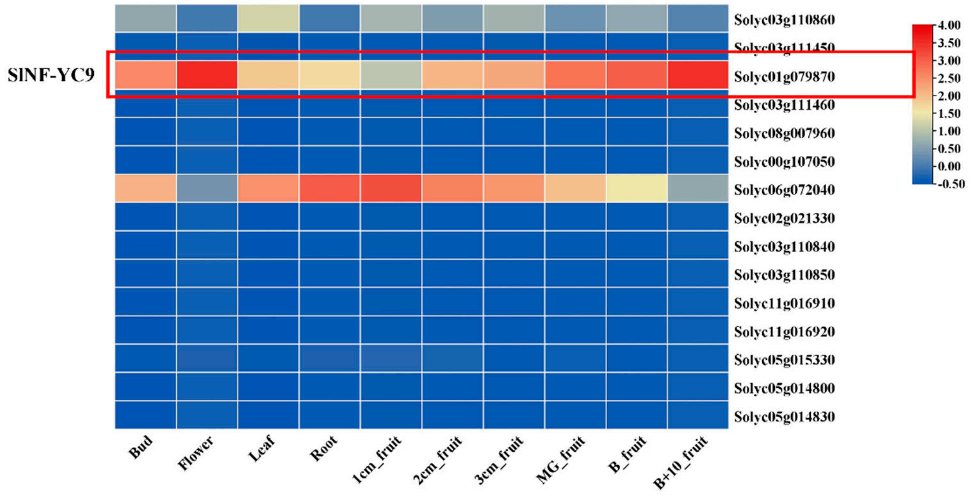

Figure S2. Heatmap visualisation showing the expression levels of SlNF-YC subfamily genes in different tissues and at different periods of fruiting of Heinz tomato varieties. The left side of the graph represents the gene name and the right side has the bordered tomato gene number. On the far right of the heatmap is a normalised scale with colours from red to blue representing high to low expression.

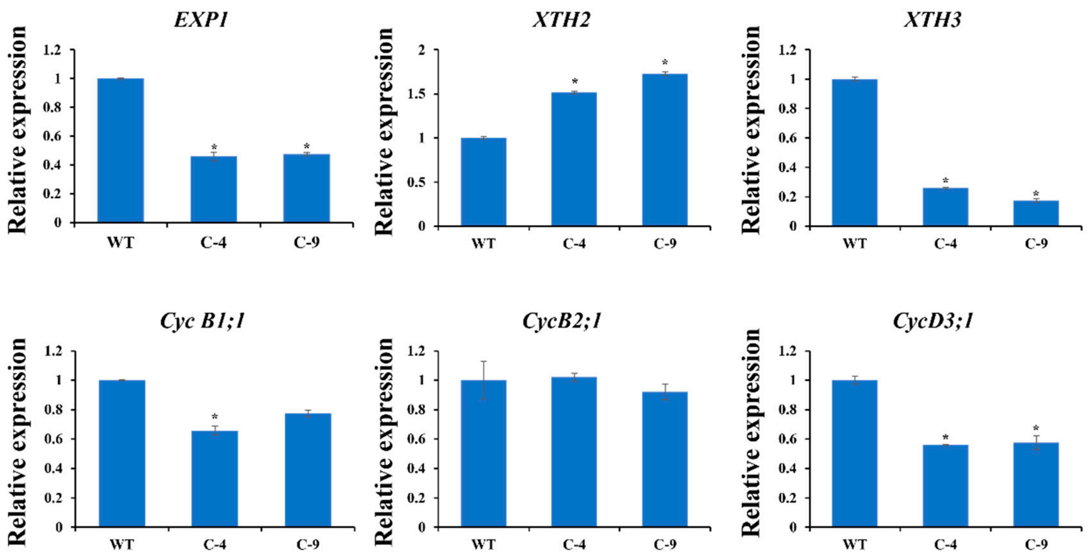

Figure S3. Analysis of the expression levels of cell division and cell cycle-related genes in *CR-slnf-yc9* and WT.

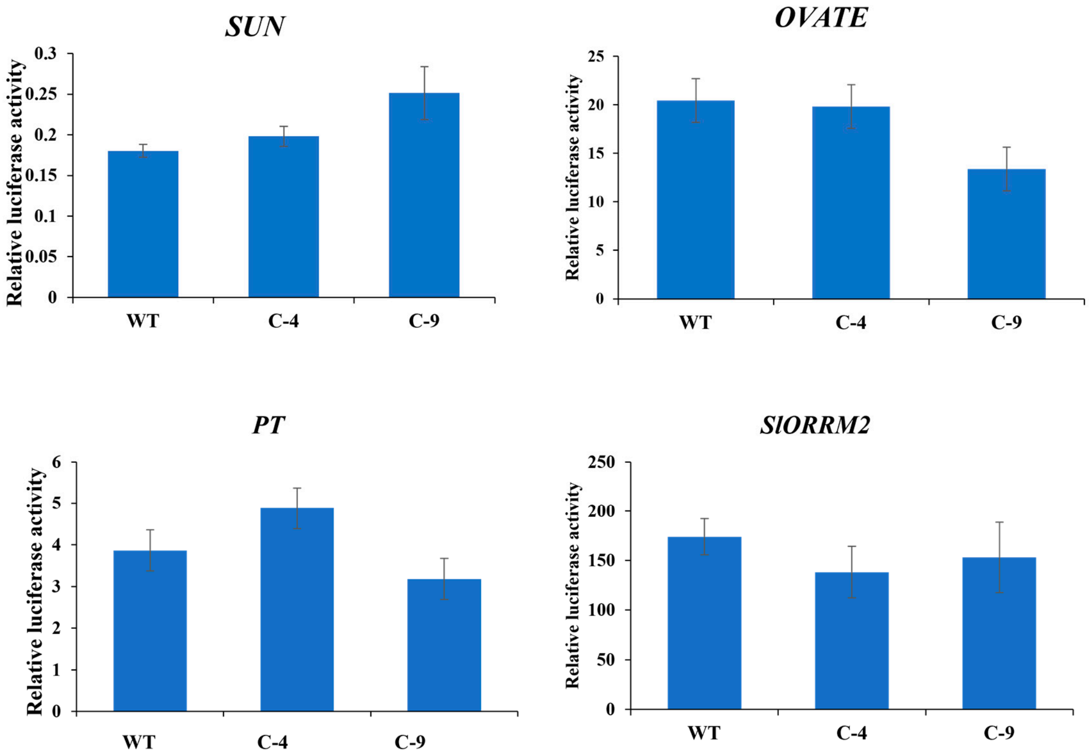

Figure S4. The expression of genes related to fruit morphogenesis in *CR-slnf-yc9* and WT.

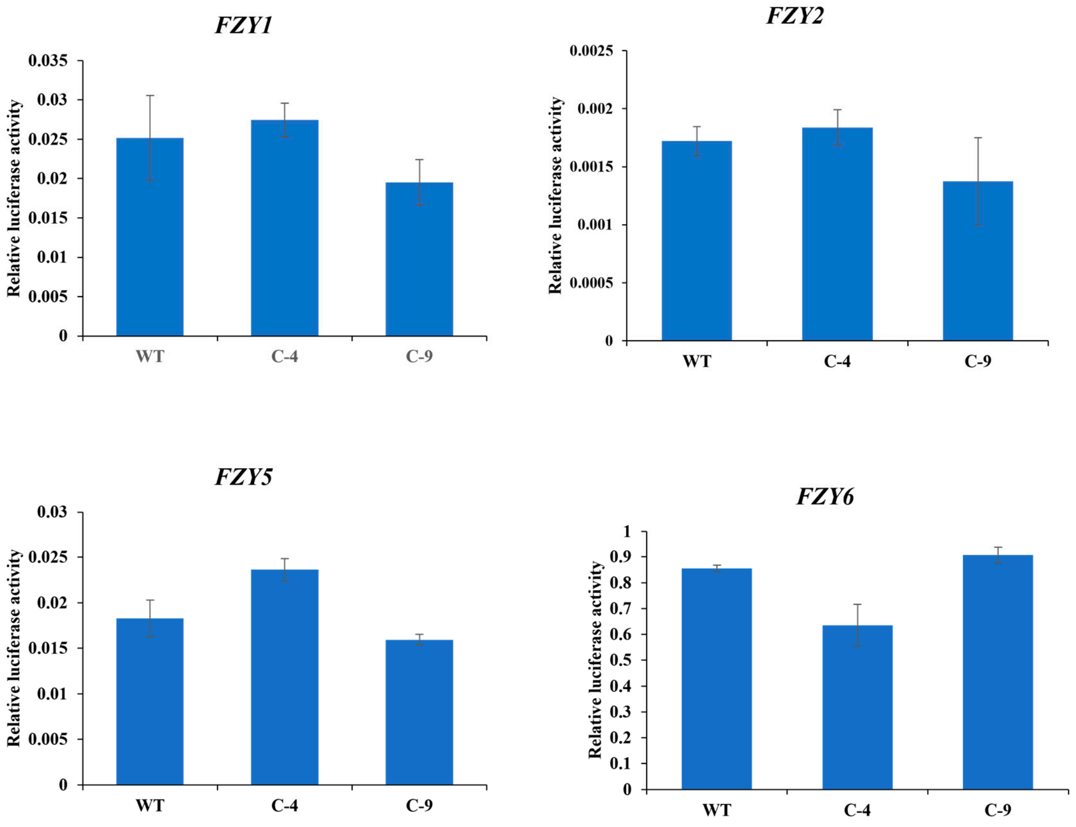

Figure S5. The expression of genes related to the rate-limiting steps of auxin synthesis in *CR-slnf-yc9* and WT.

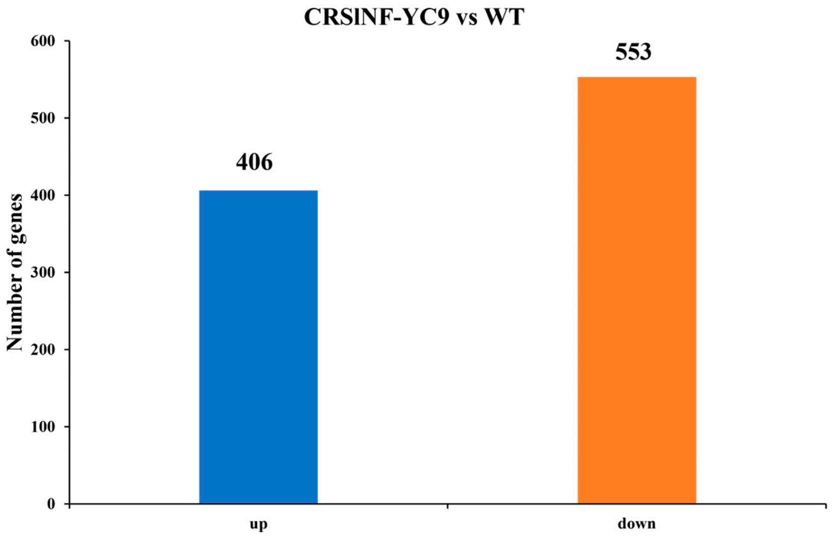

Figure S6. Number of differential genes up- and down-regulated in transcriptome sequencing in CR-slnf-yc9 compared to wild type.

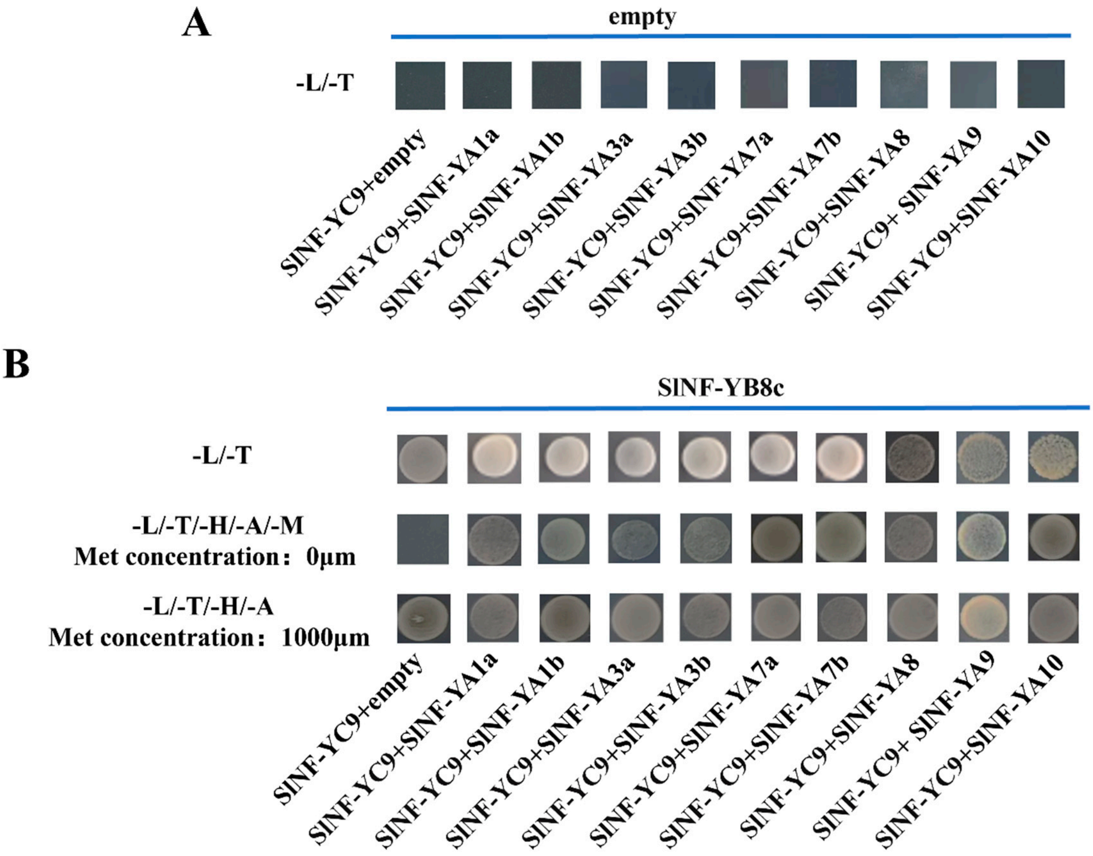

Figure S7. Yeast three-hybrid assay. **a** Negative controls were experimented with SlNF-YC9-SlNF-YAs pBridge vectors with empty BD vector. **b** Yeast three-hybrid hybridization assay to detect interactions between SlNF-YAs proteins and SlNF-YC9-Y8c heterodimers.
